# Hazardous Medications in Children with Egg, Red Meat, Gelatin, Fish, and Cow’s Milk Allergy

**DOI:** 10.3390/medicina55080501

**Published:** 2019-08-19

**Authors:** Sule Caglayan-Sozmen, Angelica Santoro, Francesca Cipriani, Carla Mastrorilli, Giampaolo Ricci, Carlo Caffarelli

**Affiliations:** 1 Division of Pediatric Allergy and Immunology,Department of Pediatrics, Faculty of Medicine, Okan University, 34722 Istanbul, Turkey; 2 Clinica Pediatrica, Azienda Ospedaliero-Universitaria, Dipartimento di Medicina e Chirurgia University of Parma, 43100 Parma, Italy; 3 Pediatric Unit, Department of Medical and Surgical Sciences, University of Bologna, 40138 Bologna, Italy; 4 UO Pediatria e Pronto Soccorso, Azienda Ospedaliero-Universitaria Consorziale Policlinico Pediatric Hospital Giovanni XXIII, 70126 Bari, Italy

**Keywords:** food allergy, children, drug hypersensitivity, IgE, oral food challenge, egg allergy, red meat allergy, gelatin allergy, fish allergy, cow’s milk allergy

## Abstract

Childhood food allergies are a growing public health problem. Once the offending food allergens have been identified, a strict elimination diet is necessary in treatment or prevention of most of the allergic reactions. Accidental food ingestion can lead to severe anaphylaxis. Food- derived substances can be used in medications at various stages of the manufacturing process. In this review, the possible roles of medications which may contain egg, red meat, gelatin, and fish allergens on allergic reactions in children with food allergy were evaluated.

## 1. Introduction

Food allergies among children have been increasing in prevalence in the last two to three decades [1]. The cornerstone of treatment for food allergy is the strict avoidance of food allergens and the use of dietary substitutions. Food allergy reactions can range from mild skin rashes to severe, potentially lethal, anaphylaxis [2]. In order to prevent such reactions, individualized advice and education on how to manage an elimination diet should be provided for parents [3]. Hidden food allergens in medications can lead to unpredictable allergic reactions. Food-derived substances are used to protect, support, or enhance stability or for bioavailability or for patient acceptability during the manufacturing process of medications [4]. The aim of this review is to provide existing data about egg, red meat, gelatin, and fish allergens in medications.

## 2. Methodology

This review focuses on food allergens in medications which physicians may have overlooked. It was obtained from searching English-language studies published during the period 1957 to 1 May 2019, in PubMed, MEDLINE. Key words/MeSH terms included: Food, allergy, and hidden. We retrieved 184 articles. Articles were considered if they described systematic reviews, or original meta-analysis, randomized-controlled trials, observational studies, and case reports on food allergens in medications in children up to 18 years of age. The titles and abstracts of the articles found were reviewed independently by two of the authors. Text of 102 publications that were considered potentially useful was assessed by searching the reference list of identified articles. We chose to cite articles of higher quality that were compatible, particularly from the view of the physician, in order to make this article more clinically important.

## 3. Hen’s Egg

### 3.1. Vaccines

Vaccines prepared on embryonated chicken eggs can contain trace amounts of egg proteins, especially ovalbumin. Chick embryo fibroblast cultures are used for measles-mumps-rubella and purified chick embryo rabies vaccines. They both contain little or no egg protein. Children who are allergic to eggs can safely receive both types of vaccines without any precautions [5].

Allergic reactions to the influenza vaccine in children with egg allergy was a matter of debate since this vaccine cultured on embryonated chicken eggs. This method theoretically could lead to higher egg protein in the vaccine. However, there is no additional risk for children with severe egg allergy [6]. Both inactivated influenza vaccine (IIV) and live attenuated influenza vaccine can be safely administered to children with egg allergy in the usual manner. The children should then be kept under medical surveillance for 30 min. Children with a previous history of immediate allergic reactions to egg that do not involve only skin (anaphylaxis) should receive the influenza vaccine under the supervision of personnel who are experts in recognizing and treating allergic reactions [7]. Yellow fever vaccine contains egg protein and children with egg allergy should receive a skin prick test to the vaccine with progressive dilution (1:10 and 1:1) and an intradermal test with a 1:100 dilution. If the test result is negative, vaccination can be given with a 60-min observation following the injection. If the skin test result is positive, the vaccine can be given in graded doses in a hospital [8]. 

### 3.2. Propofol 

Propofol is an intravenous anesthetic that is widely used for the induction and maintenance of anesthesia as well as for endoscopic and pediatric sedation. Propofol is a fat emulsion containing soybean oil and purified egg lecithin (phospholipids derived from egg yolk). The use of propofol in children with egg and soy allergy is a matter of debate. Among egg-allergic children, reactions to propofol was reported in a child with egg anaphylaxis but not in those with egg allergy [9]. Sommerfield found that two of 304 children with egg, peanut, soybean, or legume allergy experienced a possible allergic reaction to propofol that may be due to egg sensitization in children sensitized to both egg yolk and egg white. On the other hand, 124 (14%) of 892 nonallergic children met the criteria for a possible allergic reaction to propofol [10]. Use of propofol in children with eosinophlic esophagitis was evaluated in 1365 upper intestinal tract endoscopy procedures by Mehta [8]. They found no differences in the rate of allergic reactions between food-allergic and nonallergic patients. Along this line, Wiskin found no reaction to propofol in 131 children with food allergy [11] and similar findings have been reported by Asserhoj in adults [12]. Furthermore, a questionnaire survey found that propofol was given to children with egg allergy in 6 out of 26 Spanish hospitals, with no adverse reaction [13]. Overall, there is no clear evidence that allergy to egg, peanut, soybean, or legumes is a risk factor for propofol reactions and there is no definite relationship between egg allergy and severe allergic reactions to propofol. Isopropyl side chain and phenyl groups are potential allergenic epitopes of propofol. In an adult study, none of the patients allergic to propofol had food allergies. Reactions to propofol were likely due to these components. Physicians may consider prudentially avoiding propofol in patients with a history of egg anaphylaxis. Accordingly, the Childhood Allergy Committee of the Spanish Society of Allergy and Clinical Immunology recommended an alternate drug or test dose in children with a history of anaphylaxis to egg, while patients with mild egg allergy can safely receive propofol [13]. Propofol should be administered to egg-allergic patients by trained personnel who can recognize and treat anaphylaxis [12].

## 4. Red Meat

Alpha-gal is a carbohydrate antigen expressed on cells of nonprimate mammals. After a bite by the “lone star” *Amblyomma americanum* tick in USA, *Ixodes ricinus* in France, and *Ixodes holocyclus* in Australia, IgE antibodies to alpha-gal are produced. It has been shown that alpha-gal is in the intestine of the tick [14]. These antibodies were also responsible for allergy to red meat (beef, pork, or lamb) presenting as urticaria, wheezing, or anaphylaxis 3–6 h after ingestion of a mammalian meat meal. Gelatin and dairy products also contain alpha-gal. Skin prick tests with commercial meat extracts are often inconclusive. If there is a high level of suspicion, additional testing with a prick test to gelatin, prick-by-prick test with pork kidney, [15] and specific IgE to alpha-gal should be carried out [15,16].

### 4.1. Cetuximab

Sensitization to α-gal was first recognized in patients receiving cetuximab for the treatment of cancer. These patients experienced a severe, immediate, clinical hypersensitivity reaction on the first infusion of cetuximab, a chimeric monoclonal IgG antibody produced in mammalian cells. It was determined that α-gal is present on the heavy chain of the Fab portion of cetuximab. Pre-existing IgE antibodies against alpha-gal led to these reactions in patients receiving cetuximab [17,18].

### 4.2. Antivenoms 

Antivenoms are the only specific treatment for envenomation by snakebites. They are obtained from the serum of animals (horses, sheep) that have previously been immunized with the snake venom. Some antivenom preparations are enzymatically digested to produce divalent or monovalent immunoglobulin fragments (F(ab’)2/Fab) in order to reduce the total amount of the administered protein. The Fab region of these fragments include a natural amount of α-gal epitopes. Fischer et al. found equivalent reactions to antivenom and cetuximab in patients sensitized to α-gal on skin prick tests and suggested a high risk of anaphylaxis during antivenom treatment in these patients [19].

### 4.3. Prosthetic Heart Valves

Biological valves of xenogeneic origin (porcine or bovine) have been found to be associated with an increased risk of anaphylaxis in patients with α-gal allergy. The presence of α-gal in these valves induced immediate hypersensitivity reactions. It is recommended that decellularized valves should be preferred as they have no detectable α-gal [20]. A relationship between IgE antibodies to α-gal and a premature degeneration of bioprosthetic aortic valves has been suggested in two patients who developed an allergy to α-gal.

### 4.4. Recombinant Human Proteins

Recombinant human proteins (human coagulation factor VII ectapog alpha) produced in non-primate mammals may react with IgE antibodies to α-gal. A recent study showed that 5 of 9 patients sensitized to α-gal displayed a positive sIgE result for activated recombinant human coagulation factor VII (rhFVII, ectapog alpha) that is produced in baby hamster kidney cells. Recombinant human proteins should be kept in mind as a risk factor in patients with α-gal syndrome [21].

### 4.5. Heparin

Pharmaceutical-grade heparin is produced from pork (intestine) or cow and it may induce α-gal allergy. Reactions to heparin were rarely reported in patients allergic to α-gal. However, high-dose heparin derived from intestine (differs from lot to lot) is associated with an increased risk of hypersensitivity reaction [22].

### 4.6. Gelatin 

Patients who are sensitized to α-gal can develop allergic reactions to gelatins and colloids containing gelatin. However, primary gelatin allergy and α-gal allergy are different entities. Alpha-gal related gelatin-allergic patients who experienced anaphylaxis during intravenous administration of gelatin in colloid might consume 10 g of bovine gelatin orally [23]. Commercial in vitro assays for gelatin IgE showed negative results in these patients. Additional skin prick or intradermal tests should be performed with gelatin in colloids for appropriate diagnosis. A relationship between red meat, alpha-gal, and gelatin hypersensitivity has not yet been fully understood [24]. Zoster vaccine that contains gelatin may not be tolerated in some patients with allergy to alpha-gal [25,26].

## 5. Gelatin 

Gelatin is a heterogeneous mixture of peptides that is produced by procedures involving the destruction of cross-linkages between the polypeptide chains of collagen. Most medical collagen is obtained from hydrolysis of the connective tissue of animals such as cows or pigs. The protein content of gelatin may lead to allergic reactions, especially when it is administered via parenteral route.

### 5.1. Vaccines

Gelatin is added to vaccines as a preservative and stabilizer to protect the vaccine from adverse conditions such as freeze-drying or heat during storage and to maintain the vaccine as safe and effective. The amount of gelatin varies between 15 μg and more than 15,500 μg/dose of vaccine [4,27]. The highest amounts are found in the measles-mumps-rubella (MMR), rabies, varicella-zoster, oral typhoid fever, and yellow fever vaccines, and lesser amounts (up to 2000 μg/dose) in diphtheria-tetanus-pertussis (DTP) and influenza vaccines. Severe allergic reactions caused by gelatin have been described for MMR [27,28], varicella [29,30,31], yellow fever [32], and Japanese encephalitis vaccines. In the past, Japanese studies reported a high rate of positive gelatin specific IgE (86–100%) in patients with anaphylactic reactions to MMR and varicella vaccines. In contrast, U.S. and European studies revealed a lower rate of positive specific IgE (14–28%) in the same patients [27,33]. The inconsistency between results could be attributed to different types of gelatins used in the vaccines. In the 1990s, highly hydrolyzed porcine gelatin with low molecular weight was added as a stabilizer in MMR vaccines in the United States. However, partially hydrolyzed bovine gelatin with a small amount of high molecular weight was used in Japan in those years. A second explanation for the high incidence of gelatin allergy in Japan could be the existence of a genetic predisposition [5]. HLA type (DR 9) is common in Japanese patients [34]. Hydrolyzed gelatin was eliminated from the DTP vaccine in 1999. After gelatin was removed from the vaccines or converted to less allergenic highly hydrolyzed porcine gelatin, vaccine-induced anaphylaxis markedly decreased in Japan [5]. Children with red meat (bovine, pork, lamb) or milk allergy are at an increased risk of vaccine reactions due to gelatin [35]. These patients have positive specific IgE levels for bovine, lamb, pork, and milk. As we discussed previously, these patients may have detectable IgE to α-gal [36]. All children with a previous reaction to foods or vaccines containing gelatin should undergo an allergy workup. Determination of IgE to gelatin is available for diagnosis but there is no standardized skin prick extract for gelatin. A skin prick test could be an option with a prick-by-prick method melting 1 teaspoon of sugared gelatin powder in 5 mL of saline solution. If a skin test is negative but there is a history of positive gelatin allergy, an oral provocation test with gelatin should be performed. If the oral provocation test is negative, the patient should be observed for 60 min after the vaccination. If the test is positive, a gelatin-free vaccine should be administered. If that is not possible, the vaccine should be given with a graded-dose protocol [5]. If the patient is immune, an additional dose is not required but protective IgG antibody levels should be periodically confirmed [37].

### 5.2. Gelatin-Based Colloids

In patients with hypotension, circulating blood volume is commonly increased by colloid plasma volume expanders. The type of colloid varies between countries. In UK, gelatin-based solutions have been traditionally administered, while starches based on colloids and albumin are used almost exclusively in Switzerland and in the United States, respectively [38]. Gelatin-based solutions are heterogeneous mixtures of polypeptides, usually produced by hydrolysis of bovine collagen, including large amounts of proline and hydroxyproline residues [39]. Albumin solution is derived from donated blood by fractionation and/or plasmapheresis. The processing is expensive [40]. Hence, gelatin- (1.7% of all fluid administrations) and starch-based (0.2%) solutions are more widely used than albumin (0.1%) [41]. The incidence of anaphylaxis to gelatin solutions is 6.2 per 100,000 doses and anaphylaxis occurs within 10 min after administration [42]. However, in 11 out of 12 adult patients with anaphylaxis to intravenous gelatin-based solutions, only three patients showed immediate reactions, within 5 min, after the administration of a solution, while most of the reactions occurred 10–70 min later [43]. All of them suffered from severe allergic reactions and three of them developed cardiac arrest. Skin prick tests and intradermal tests (when the SPT was negative or indeterminate) confirmed allergy in 11 patients and an intravenous provocation test was positive in one patient with a negative skin test. Evidence supporting the use of gelatin-based colloids for intraoperative hypotension is lacking and the risk of life-threatening allergic reactions to gelatin solutions is higher in comparison with other plasma expanders, so their use should be reconsidered [43]. 

### 5.3. Other Drugs

Suppositories. Systemic allergic reaction developed in 10 children with a chloral hydrate rectal suppository containing gelatin. Five of these children had anaphylaxis and all of them had positive specific IgE to gelatin [44].

### 5.4. Erythropoietin

A single case reported anaphylaxis to a gelatin stabilizer in the erythropoietin product after intravenous injection. She had positive antibovine gelatin IgE and tolerated the erythropoietin product containing human albumin as a stabilizer [45].

Gelatin based hemostatic products. Topical gelatin-based hemostatic products have been shown to induce allergic reactions in children during spinal surgery, dental treatment, and liver biopsy [46,47,48,49]. Even in patients who could tolerate gelatin-containing food and vaccines, a severe allergic reaction can develop due to the different route of administration [49].

Available data do not report that patients allergic to fish react to fish gelatin that is contained in an allergen extract of grass pollen from Timothy (*Phleum pratense*) oral lyophilisate. However, treatment in patients with fish allergy should be cautiously started.

## 6. Fish

Protamine was initially isolated from salmon sperm and currently produced by recombinant technology. Protamine is used to counteract the anticoagulant properties of heparin or to delay insulin absorption in slow-release preparations of insulin. Transient systemic hypotension, anaphylaxis, and severe pulmonary vasoconstriction can occur after protamine administration [50]. These protamine-related adverse reactions attributed to fish allergy are due to a possible cross reactivity between fish and protamine. However, the evidence supporting this hypothesis is lacking. Inhibition of IgE binding to salmon with protamine sulphate or the presence of serum IgE to protamine in enzyme-linked immunosorbent assay (ELISA) have not been shown [51]. Levy et al. determined that fish allergy is not a risk factor for protamine reactions [52]. Protamine reactions in patients using insulin have been reported. Recently, three cases of protamine allergy were evaluated and two of them subsequently received insulin without any reaction. Other mechanisms, rather than allergy, may lead to these reactions [53].

Fish-allergic patients can receive both protamine and insulin without any precaution.

Fish oils are generally highly purified and should not contain fish protein. Fish-allergic patients can consume fish oils uneventfully [4,54].

## 7. Cow’s Milk

Cow’s milk proteins can contaminate lactose that is used as a pharmaceutical excipient. The prevalence of reactions to cow’s milk proteins in medications has not been investigated. It appears to be low, even if increasing.

### 7.1. Corticosteroids

Cow’s milk proteins can contaminate lactose contained in fluticasone/salmeterol or laninamivir dry-powder inhalers (DPI) and rarely elicit anaphylaxis in children with cow’s milk allergy (CMA) [55]. However, patients allergic to milk usually tolerate lactose in DPI [44]. Therefore, it is suggested to be aware when such drugs are given to children with CMA.

Lactose is also contained in 40 mg methylprednisolone sodium succinate for injections. This methylprednisolone formulation can be contaminated by milk proteins in the lactose vehicle. Consequently, an intravenous methylprednisolone sodium succinate 40 mg injection provoked anaphylactic reactions or urticaria in children with severe CMA [56,57,58,59]. Noteworthy, skin tests were positive to methylprednisolone sodium succinate 40 mg injection and negative to lactose-free methylprednisolone formulation [56,57,58,59]. The European Medical Agency has contraindicated intravenous methylprednisolone sodium succinate 40 mg in children with CMA or suspected CMA [60].

### 7.2. Vaccines

Case reports have described clinical hypersensitivity reactions caused by diphtheria-tetanus-pertussis vaccine in children with CMA [61], potentially elicited by caseins in culture media [62]. The oral polio vaccine may contain alpha-lactalbumin and its administration may trigger hypersensitivity reactions in children allergic to milk [63]. However, these findings have not been confirmed by adverse events recorded in the Vaccine Adverse Event Reporting System database [62]. However, children with CMA should be cautiously kept under surveillance for 1 h after they receive the diphtheria-tetanus-pertussis vaccine or oral polio vaccine.

## 8. Other Drugs

Hidden cow’s milk proteins have been detected in probiotics [64]. Although adverse reactions to probiotics are sparsely reported, anaphylaxis has been described in a patient with CMA [65,66]. Lactulose is made from lactose and it may be contaminated by milk proteins. A case report described the onset of a dry cough and wheezing associated with ingestion of lactulose syrup for the treatment of constipation in a 4-year-old child with severe CMA. An oral challenge was performed. After a total of 2 mL of lactulose, the child presented with oral itching, diffuse erythema with conjunctival hyperemia, sneezing, and coughing followed by wheezing [67]. 

Overall, even though cow’s milk-allergic children can tolerate lactose-containing products, the risk of developing reactions should be considered. It is necessary to include a warning label about residual allergen content in the medications to prevent allergic hypersensitivity reactions in patients with CMA.

## 9. Conclusions

This review provides detailed data for the avoidance of egg, red meat, gelatin, fish, and cow’s milk allergens in medications. Patients who have an allergy should be informed of the ingredients in medications, even for exposure to trace amounts of food allergens. Table 1 summarizes the medications mentioned as food allergens.

## Figures and Tables

**Table 1:** Summary of medications having mentioned food allergens.

Offending Allergen	Medication	Ingredient	Recommendations for Administering Medications	Reference	Diagnosis
Hen’s egg	Measles-mumps-rubella (MMR) vaccine	Vaccines are grown in chick embryo fibroblast cultures	Administer in the usual manner	[5]	There is no need for an additional test
	Influenza vaccine (live and attenuated)	Vaccine antigens are prepared in chicken eggs	Administer in the usual manner in general practitioner office, keep under observation for 30 min Administer in allergist’s office if there is a history of anaphylaxis to egg	[6,7]	There is no need for an additional test
	Yellow fever vaccine	Vaccine contains egg protein	Perform SPT prior to administration	[8]	SPT If negative–Administer and observe 60 min afterwards If positive–Administer in graded doses at hospital
	Rabies vaccine	Vaccine is grown in chick embryo fibroblast cultures	Administer in the usual manner	[5]	There is no need for an additional test
	Propofol	Contains purified egg lecithin	Children with mild allergy—Administer in the usual manner by personnel recognized to treat anaphylaxis Children with a history of anaphylaxis to egg—Choose an alternate drug or apply a test dose prior to administration	[12,13]	It is not recommended to perform skin tests with propofol before administering it.
Red meat (alpha-gal allergy)	Cetuximab	Fab portion has alpha-gal antigen	Severe allergic reaction can be seen after administration. If cetuximab is necessary for treatment, a desensitization protocol should be given in a hospital	[17,18]	Prick-by-prick test with pork kidney Specific IgE to alpha-gal
	Antivenom for snake bites	Immunoglobulin fragments have alpha-gal antigen	High risk of anaphylaxis	[19]	Prick-by-prick test with pork kidney Specific IgE to alpha-gal
	Prosthetic heart valves	Valves contain alpha-gal	Premature degeneration Use decellularized valves	[20]	Prick-by-prick test with pork kidney Specific IgE to alpha-gal
	Recombinant human protein (human coagulation factor VII)	Contain alpha-gal allergen	Typical allergic symptoms (urticaria, angioedema, bronchospasm)	[21]	Prick-by-prick test with pork kidney Specific IgE to alpha-gal
	Heparin	May contain alpha-gal allergen	Heparin derived from intestine can lead to hypersensitivity reactions	[22]	Prick-by-prick test with pork kidney Specific IgE to alpha-gal
Gelatin	Measles-mumps-rubella (MMR), rabies, varicella-zoster, oral typhoid fever and yellow fever vaccines	High amounts of gelatin content	All children with a history of gelatin allergy should be evaluated by an allergist Alpha-gal allergy should be kept in mind (see text)	[27,28,29,30,31,32,33,34,35,36,37]	Specific IgE to gelatin If it is not possible, perform SPT with gelatin powder (see text) In case of positive results, choose gelatin-free vaccines or administer vaccine with a graded-dose protocol
	DTP and influenza	Low amounts of gelatin content			
	Plasma volume expanders	Gelatin-based colloids	Severe allergic reactions can occur during infusion Prefer albumin- and starch-based solutions, if needed	[38,39,40,41,42,43]	Gelatin-specific IgE SPT and intradermal test
	Suppositories	Contain gelatin	May lead to anaphylaxis	[44]	Gelatin-specific IgE SPT and intradermal test
	Erythropoietin	Contain gelatin stabilizer	May lead to anaphylaxis (single case)	[45]	Gelatin-specific IgE SPT and intradermal test
	Hemostatic products	Gelatin-based	Allergic reactions during dental treatment or surgery	[49]	Gelatin-specific IgE SPT and intradermal test
Fish	Protamine	Produced from salmon sperm	Administer in the usual manner	[52,53]	There is no need for an additional test
	Insulin	Contain protamine	Administer in the usual manner		There is no need for an additional test
Cow’s milk	Corticosteroids	Dry-powder inhalers (DPI) (fluticasone/salmeterol or laninamivir) can contain lactose	Rarely induce allergic reactions Administer in the usual manner	[55]	There is no need for an additional test
	Corticosteroids	40 mg methylprednisolone sodium succinate	Provoke anaphylactic reactions Contraindicated intravenous methylprednisolone sodium succinate 40 mg in children with CMA or suspected CMA	[56,57,58,59,60]	There is no need for an additional test
	Diphtheria-tetanus-pertussis	Contain caseins in culture media	Administer in the usual manner, observe one hour after vaccination	[61,62]	There is no need for an additional test
	Oral polio vaccine	May contain alpha lactalbumin	Administer in the usual manner, observe one hour after vaccination	[63]	There is no need for an additional test
	Probiotics	May contain lactose	Rare allergic reaction Administer in the usual manner	[65,66]	There is no need for an additional test
	Medications for constipation	May contain lactulose	Risky in children with severe CMA (case report)	[67]	There is no need for an additional test
